# Association Between Different Insulin Resistance Indices and Heart Failure in US Adults With Diabetes Mellitus

**DOI:** 10.1111/anec.70035

**Published:** 2024-11-15

**Authors:** Lirong Chen, Lin Qian, Yongming Liu

**Affiliations:** ^1^ The First Clinical Medical College of Lanzhou University Lanzhou Gansu China; ^2^ Department of Geriatric Cardiology The First Hospital of Lanzhou University, Gansu Provincial Clinical Research Center for Geriatric Diseases Lanzhou Gansu China

**Keywords:** cardiovascular metabolism, diabetes, heart failure, insulin resistance, metabolic score for insulin resistance

## Abstract

**Purpose:**

This study aims to scrutinize the association between various Insulin Resistance (IR) indices and heart failure (HF) risk in adult diabetics within the United States.

**Methods:**

The National Health and Nutrition Examination Survey (NHANES) (2005–2018) dataset was used in this study. Weighted logistic regression analysis and restricted cubic spline were employed to ascertain the correlation between IR indices and the incidence of HF in diabetic patients. The predictive capability of the IR indices was evaluated using the Receiver Operating Characteristic curve.

**Results:**

This study included a total of 2574 diabetic patients, out of which 209 (8.1%) were diagnosed with HF. After the adjustment of potential confounders, TyG‐BMI (OR: 1.005, 95% CI: 1.002–1.009), TG/HDL‐C (OR: 1.138, 95% CI: 1.024–1.265), and METS‐IR index (OR: 1.035, 95% CI: 1.015–1.057) were significantly associated with HF risk. RCS curves revealed nonlinear dose–response relationship between TyG, TyG‐BMI, TG/HDL‐C, and the occurrence of HF in diabetic patients. Subgroup analyses showed that four IR indices were positively associated with the risk of HF in the elderly diabetic population. Unfortunately, all IR indices failed to improve the predictive performance of the underlying risk model for HF in diabetic patients.

**Conclusion:**

Four IR markers may be important predictors of HF risk in diabetics.

## Graphical abstract

Our main objective was to investigate the relationship between different insulin resistance indices and the risk of heart failure in US adults with diabetes, with a view to identifying patients at high risk of heart failure in the diabetic population at an early stage of the disease and intervening early to reduce the incidence of heart failure and improve patient prognosis. In conclusion, we found a U‐shaped dose–response relationship between TyG, TyG‐BMI, TG/HDL‐C, and heart failure risk in diabetic patients, whereas METS‐IR was linearly correlated. This study has potential implications for secondary prevention and stratification of heart failure risk in diabetic patients.

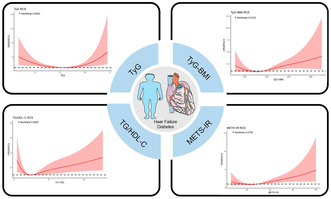

**Abbreviations**
AGEs: advanced glycation end products
AUC: area under curve
BMI: body mass index
BUN: urea nitrogen
CAD: coronary artery disease
CHD: coronary heart disease
CI: confidence interval
DBP: diastolic blood pressure
DM: diabetes mellitus
eGFR: estimated glomerular filtration rate
FPG: fasting glucose
HbA1c: glycated hemoglobin
HDL‐C: high‐density lipoprotein cholesterol
HF: heart failure
ICM: ischemic cardiomyopathy
IDI: integrated discrimination improvement
IQR: interquartile range
IR: insulin resistance
LDL‐C: low‐density lipoprotein cholesterol
MACCEs: major adverse cardiac and cerebrovascular events
MEC: mobile examination center
METS‐IR: metabolic score for insulin resistance
MR: mendelian randomization
NCHS: national center for health statistics
NHANES: national health and nutrition examination survey
NRI: Net reclassification improvement
OR: odds ratio
RCS: restricted cubic spline
ROC: receiver operating characteristic curve
ROS: reactive oxygen species
SBP: systolic blood pressure
SD: standard deviation
T2DM: type 2 diabetes
TC: total cholesterol
TG: total triglyceride
TG/HDL‐C: triglyceride to high‐density lipoprotein cholesterol ratio
TyG: triglyceride glucose
TyG‐BMI: triglyceride glucose with body mass index
UA: uric acid
UP: urine protein

## 1 Introduction

Heart failure (HF) is a global health issue characterized by high morbidity and mortality rates, with an estimated global prevalence of 1% to 2%, affecting over 60 million individuals worldwide (Becher et al. 2022; Seferović et al. 2021). A notable comorbidity is the co‐occurrence of type 2 diabetes mellitus (T2DM) and HF, where approximately one‐third of HF patients also suffer from diabetes. Diabetes increases the risk of developing HF by two to five times (Pop‐Busui et al. 2022).

Insulin resistance (IR) plays a critical role in the development and progression of T2DM and is defined as a condition where insulin secretion remains normal, but the ability of tissues, organs, and cells to respond to insulin is diminished. This impairs insulin's function in promoting glucose uptake and utilization in various organs (Bornfeldt and Tabas 2011). T2DM is closely linked to IR, as IR is central to its onset and progression (Altaf, Barnett, and Tahrani 2015). Recent research has shown a significant correlation between increased IR and the incidence of HF (Yan et al. 2019). IR contributes to HF by inducing cardiomyocyte damage, myocardial fibrosis, and myocardial hypertrophy through mechanisms such as oxidative stress, disrupted insulin signaling pathways, and increased inflammation in cardiomyocytes (Wang et al. 2022). This creates a vicious cycle of exacerbating HF and other comorbidities like T2DM, leading to worsened clinical outcomes, including increased morbidity, mortality, readmission rates, and disability (Shaw and Cooper 2020).

Given the detrimental effects of IR on HF, early and accurate identification of IR is crucial. However, the gold‐standard method for assessing IR, the hyperinsulinemic‐euglycemic clamp procedure, is complex and impractical for widespread use in clinical and epidemiological studies (Muniyappa et al. 2008). As a result, alternative, more accessible markers of IR are needed.

One promising surrogate marker for IR is the triglyceride glucose (TyG) index, which is calculated from fasting triglyceride and glucose levels. Several studies have demonstrated that the TyG index is associated with the development and prognosis of HF. For example, a cohort study identified a J‐shaped relationship between the TyG index and HF risk (Xu et al. 2022), while another study found that a higher TyG index correlates with increased HF incidence and impaired left ventricular structure and function (Huang et al. 2022). In patients with acute decompensated HF, an elevated TyG index was independently associated with higher risks of all‐cause death, cardiovascular death, and major adverse cardiac and cerebral events. Furthermore, in patients with chronic HF, higher TyG index tertiles were linked to an increased risk of mortality, particularly in those with metabolic syndrome and preserved ejection fraction (Zhou et al. 2023). These findings highlight the potential of the TyG index as a valuable predictor for HF risk stratification and prognosis. The TyG‐BMI index, which integrates body mass index (BMI) into the TyG calculation, has emerged as an even stronger predictor of IR than the TyG index alone (Lim et al. 2019). Studies have shown that TyG‐BMI is more closely associated with coronary artery disease (CAD) severity than TyG, although the metabolic score for insulin resistance (METS‐IR) demonstrates the highest predictive value (Zhang et al. 2022). The inclusion of BMI in the TyG calculation enhances its ability to reflect metabolic disorders and obesity‐related IR, which are important contributors to cardiovascular disease progression (Er et al. 2016). The TG/HDL‐C ratio has also been identified as a marker of lipid metabolism dysregulation and has been linked to CAD, a major risk factor for HF in diabetic patients. More recently, non‐insulin‐based IR indices, such as the TyG index, TyG‐BMI, TG/HDL‐C ratio, and METS‐IR, have gained attention for their potential to predict cardiovascular outcomes, including HF and CAD severity. For instance, elevated TyG index and TG/HDL‐C ratio have been associated with increased HF prevalence in overweight/obese adults without diabetes (Cui et al. 2024). Additionally, the TyG index demonstrated a J‐shaped relationship with HF incidence in a large cohort study (Xu et al. 2022). In the context of predicting CAD severity, METS‐IR had the highest predictive value, followed by TyG‐BMI. Importantly, for predicting 5‐year mortality in critically ill patients with chronic HF, the TyG index outperformed both TyG‐BMI and the TG/HDL‐C ratio (Zhou et al. 2024).

This study aims to explore the association between non‐insulin‐based IR indices and the risk of HF in adults with diabetes, contributing to a better understanding of the metabolic pathways leading to HF and enabling more effective risk stratification. Specifically, the research will focus on four surrogate IR indices—TyG, TyG‐BMI, TG/HDL‐C ratio, and METS‐IR (Simental‐Mendía, Rodríguez‐Morán, and Guerrero‐Romero 2008)—to evaluate their prognostic value in predicting HF risk in diabetic patients. By utilizing easily accessible clinical variables like triglycerides, glucose, and BMI, this study seeks to offer a feasible approach to early HF risk stratification and provide valuable insights for both primary and secondary prevention strategies in clinical practice.

## 2 Methods

### 2.1 Data Sources

**FIGURE 1:**
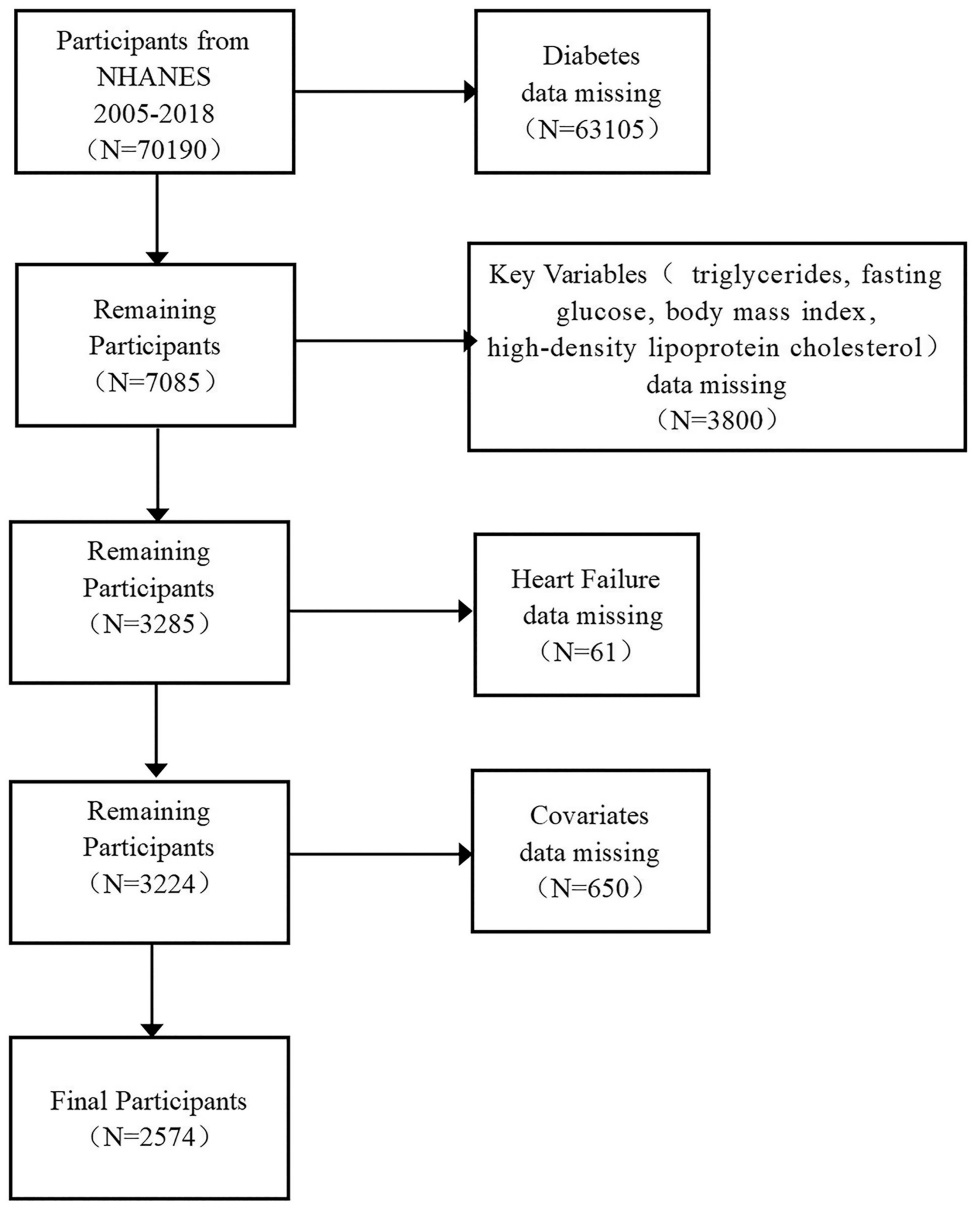
The flow chart of the systematic selection process.

Data were obtained from the National Health and Nutrition Examination Survey (NHANES), a nationally representative cross‐sectional survey of the health and nutritional status of the US population living in institutions. The study employs a sophisticated, hierarchical, multistage probabilistic clustering design model. The database is accessible at https://www.cdc.gov/nches/nhanes. The NHANES protocol received approval from the National Center for Health Statistics (NCHS) Research Ethics Review Board, and all participants provided signed informed consent forms. The study population consisted of 2574 patients with diabetes mellitus from 2005 to 2018. The exclusion criteria were as follows: (1) individuals under the age of 18 years and those with missing diabetes data; (2) subjects who had missing data on key variables, such as total triglycerides (TG), fasting glucose (FPG), body mass index (BMI), high‐density lipoprotein cholesterol (HDL‐C) indices, and other relevant covariates; (3) patients who either were unaware of their HF status or declined to disclose this information. Detailed descriptions of the sampling and exclusion criteria can be found in Figure 1.

### 2.2 Definitions of TyG, TyG‐BMI, TG/HDL‐C, and METS‐IR Score

The calculation of the non‐insulin basal IR index was executed utilizing the subsequent mathematical expressions (Simental‐Mendía, Rodríguez‐Morán, and Guerrero‐Romero 2008):

$\mathrm{TyG}=\ln\left[\mathrm{TG}\;\left(\mathrm{mg}/\mathrm{dL}\right)\times \mathrm{FPG}\;\left(\mathrm{mg}/\mathrm{dL}\right)/2\right]$

TyG‐BMI=TyG×BMI

$\mathrm{TG}/\mathrm{HDL}‐C=\mathrm{TG}\;\left(\mathrm{mg}/\mathrm{dL}\right)/\mathrm{HDL}‐C\;\left(\mathrm{mg}/\mathrm{dL}\right)$

$\text{METS}‐\mathrm{IR}=\ln\;\left[\left(2\times \mathrm{FPG}\;\left(\mathrm{mg}/\mathrm{dL}\right)\right)+\mathrm{TG}\;\left(\mathrm{mg}/\mathrm{dL}\right)\right]\times \mathrm{BMI}/\ln\;\left(\mathrm{HDL}‐C\;\left(\mathrm{mg}/\mathrm{dL}\right)\right)$

### 2.3 The Diagnosis of Diabetes and HF

Diabetes was defined as any of the following: (1) HbA1c ≥ 6.5%; (2) fasting glucose ≥ 126 mg/dL; (3) self‐reported diagnosis of diabetes; (4) self‐reported use of insulin or other diabetes medication. Congestive HF was diagnosed based on patients' self‐reported diagnosis from their physicians (physicians diagnosed congestive HF).

### 2.4 Covariates

The covariates incorporated in this study included gender, age, race, smoking history, alcohol consumption history, physical activity (categorized as vigorous or moderate), dietary pattern (measured by dietary protein intake, carbohydrate intake, total sugar intake, total fat intake, and cholesterol intake), total cholesterol (TC), low‐density lipoprotein cholesterol (LDL‐C), urine protein (UP), uric acid (UA), urea nitrogen (BUN), estimated glomerular filtration rate (eGFR), chronic diseases (hypertension, stroke, coronary heart disease (CHD), liver condition), and antihyperglycemic drugs. The data pertaining to gender, age, and race were sourced from the NHANES interview. Smoking status was bifurcated into two categories: infrequent smokers (those who had smoked less than 100 cigarettes in their lifetime) and regular smokers (those who had smoked 100 or more cigarettes). Alcohol consumption status was further divided into two groups: infrequent drinkers (those who averaged less than one drink per day over the past 12 months) and frequent drinkers (those who consumed alcohol at least once daily on average over the same period). Vigorous physical activity was defined as activities that caused heavy sweating or a significant increase in respiration or heart rate, and moderate physical activity was defined as activities that caused light sweating or a mild to moderate increase in respiration or heart rate. Dietary protein intake, carbohydrate intake, total sugar intake, total fat intake, and cholesterol intake were obtained from the dietary section of the database. Dietary recall interviews were conducted at a mobile examination center (MEC), and participants' total nutrient intake on day one was used for this study. After a rest period of more than 5 min, systolic blood pressure (SBP) and diastolic blood pressure (DBP) were measured three times by trained healthcare personnel using a mercury sphygmomanometer. The arithmetic mean of these three blood pressure measurements, expressed in millimeters of mercury (mmHg), was used for subsequent analysis. Blood samples were collected in a mobile examination unit and stored at 20°C before being sent to the central laboratory for measurement using standard methods. A history of CHD, stroke, and liver disease was defined as a previous diagnosis of CHD, stroke, and liver disease, respectively. Hypertension was diagnosed if SBP ≥ 130 mmHg or DBP ≥ 80 mmHg, use of antihypertensive medications, or a history of hypertension. BMI was calculated as weight (kg) divided by the square of height (m). eGFR was calculated as [(140−age) × weight (kg)] × 0.85 (if female)/[72 × serum creatinine (mg/dL)] (Levey et al. 2006).

### 2.5 Statistical Analysis

All statistical analyses were conducted using R Studio version 4.2.2, and we applied the NHANES complex sampling design throughout the analysis. Sample weights, strata, and primary sampling units (clusters) were applied to account for the multistage, stratified probability sampling used by NHANES. The application of these weights ensures that our results are representative of the US population. We used the appropriate NHANES sample weights based on the combination of multiple years of data (2005–2018) and the specific variables included in the analysis. The survey weights were applied using the survey package in R, and weighted logistic regression models were employed to examine the association between the IR indices and HF risk in diabetic patients. Sample weights were diligently incorporated in conformity with the NHANES analysis guidelines, ensuring due consideration of the intricacies inherent to the survey design (Gu et al. 2012). Continuous variables conforming to a normal distribution are displayed as mean ± standard deviation (SD). Conversely, continuous variables that exhibit a non‐normal distribution are displayed as median (interquartile range), and categorical variables are delineated by their median values along with the interquartile range (IQR). Categorical variables, on the other hand, are expressed as numbers and weighted proportions. Multivariate weighted logistic regression models were systematically formulated by adjusting for distinct covariates, to investigate the relationship between IR indices and HF in diabetic patients. The findings are succinctly presented as the odds ratios (ORs) and 95% confidence intervals (CIs). Model 1 was left unadjusted, whereas Model 2 was adjusted for factors such as age, gender, race, smoking history, and alcohol consumption history. Model 3, which further refined the adjustments made in Model 2, was then adjusted for variables including physical activity (vigorous, moderate), dietary pattern (dietary protein intake, carbohydrate intake, total sugar intake, total fat intake, cholesterol intake), total cholesterol (TC), low‐density lipoprotein cholesterol (LDL‐C), urine protein (UP), uric acid (UA), urea nitrogen (BUN), estimated glomerular filtration rate (eGFR), chronic diseases (hypertension, stroke, CHD, liver condition), and antihyperglycemic drugs. Furthermore, we employed multivariable‐adjusted restricted cubic spline regression models to examine the potential linear or nonlinear correlation between IR indices and HF risk in diabetic patients. We selected three knots for the spline models, following commonly used practices in epidemiological research. The knots were placed at the 10th, 50th, and 90th percentiles of the distribution of the independent variables (the IR indices) to capture the nonlinear relationship between the IR indices and HF risk. This approach allows for sufficient flexibility in modeling potential nonlinear associations while avoiding overfitting, which can occur with an excessive number of knots. We chose these percentiles to balance precision in modeling the relationship across the entire data range, particularly at the extremes, while maintaining model interpretability and robustness. Sampling weights were applied in the restricted cubic spline regression models to account for the complex survey design of NHANES. This ensures that the results are representative of the US population and adjusts for the stratified, clustered nature of the dataset. The application of weights in the spline models allows us to produce valid and generalizable estimates of the nonlinear relationship between the IR indices and the risk of HF in diabetic patients, consistent with the weighting approach used throughout the rest of the analysis. The predictive accuracy of these various IR indices for assessing HF risk in diabetic patients was assessed using the area under the ROC curve (DeLong, DeLong, and Clarke‐Pearson 1988). To investigate potential differences in IR indices among sub‐populations of diabetic patients, we conducted stratification based on key demographic and clinical factors. These factors included gender, age (< 60 vs. ≥ 60 years), smoking history (yes vs. no), alcohol consumption history (yes vs. no), history of liver disease (yes vs. no), and history of hypertension (yes vs. no). We also considered the potential influence of diabetes duration and lipid‐lowering medication on our results. To examine this, we performed a sensitivity analysis that incorporated diabetes duration and lipid‐lowering medication as potential confounders. This was done after excluding 1780 individuals with missing data on lipid‐lowering medication and diabetes duration, allowing us to rerun a weighted logistic regression.

## 3 Results

### 3.1 Baseline Characteristics of Study Participants

Baseline characteristics of the included patients are shown in Table 1. The study population consisted of 2574 eligible participants, including 209 (8.1%) patients with HF and 2365 (91.9%) patients without heart failure. The median age was 61 years (IQR, 51–69). Non‐Hispanic white individuals comprised 63% of the study population, and males constituted 51% of the total cohort. Hypertension, CHD, stroke, and glucose‐lowering medication use were more prevalent among the included patients. In comparison between the HF and non‐HF groups, there were significant differences in age, alcohol consumption, vigorous physical activity, TC, HDL‐C, LDL‐C, BUN, eGFR, UP, UA, BMI, TG/HDL‐C, METS‐IR (*p* < 0.05).

**TABLE 1:** Baseline characteristics of subjects.

Characteristic	Overall, *N* = 2574	Non‐HF, *N* = 2365	HF, *N* = 209	*p*
Age(years)	61 (51, 69)	60 (51, 69)	69 (61, 77)	< 0.001
Gender(*n*, %)				
Male	1339 (51%)	1225 (51%)	114 (52%)	0.770
Female	1235 (49%)	1140 (49%)	95 (48%)	
Race (*n*, %)				
Mexican American	417 (8.5%)	394 (8.7%)	23 (6.1%)	0.316
Other Hispanic	283 (5.9%)	262 (6.1%)	21 (3.5%)	
Non‐Hispanic White	965 (63%)	866 (63%)	99 (65%)	
Non‐Hispanic Black	655 (15%)	597 (14%)	58 (19%)	
Other race	254 (8.1%)	246 (8.2%)	8 (7.2%)	
Alcohol consumption (*n*, %)	1321 (56%)	1235 (57%)	86 (46%)	0.026
Smoking status (*n*, %)	1261 (50%)	1148 (50%)	113 (52%)	0.729
Physical activity (*n*, %)				
Vigorous	231 (9.7%)	220 (10%)	11 (4.0%)	0.011
Moderate	874 (36%)	823 (36%)	51 (29%)	0.157
Dietary pattern				
Protein (g)	71 (51,96)	72 (52, 96)	62 (46, 96)	0.073
Carbohydrate (g)	203 (150, 278)	204 (150, 280)	196 (142, 254)	0.092
Total sugars (g)	76 (47, 123)	77 (47, 122)	75 (50, 126)	0.886
Total fat (g)	70 (48, 102)	70 (48, 102)	70 (39, 100)	0.388
Cholesterol (mg)	235 (134, 402)	236 (135, 403)	203 (112, 381)	0.232
Laboratory results				
TC (mg/dL)	177 (152, 208)	178 (153, 208)	166 (136, 190)	0.009
TG (mg/dL)	130 (90, 182)	130 (90, 182)	142 (90, 190)	0.317
HDL‐C (mg/dL)	46 (40, 56)	46 (40, 56)	43 (37, 51)	0.003
LDL‐C(mg/dL)	99 (78, 126)	100 (78, 127)	89 (69, 115)	0.015
FPG (mg/dL)	134 (121, 166)	134 (122, 166)	131 (116, 163)	0.421
BUN (mg/dL)	14 (11, 19)	14 (11, 18)	19 (14, 26)	< 0.001
eGFR (mL/min)	107 (79, 144)	109 (81, 147)	78 (55, 118)	< 0.001
UP (ug/mL)	13 (6, 31)	12 (6, 29)	22 (8, 68)	0.002
UA (mg/dL)	5.7 (4.80, 6.70)	5.6 (4.80, 6.70)	6.34 (5.00, 8.00)	< 0.001
BMI (kg/m^2^)	32 (28, 37)	32 (28, 37)	34 (29, 39)	0.020
History of disease, *n* (%)				
Hypertension	2040 (78%)	1849 (76%)	191 (94%)	< 0.001
CHD	244 (9.6%)	162 (7.3%)	82 (38%)	< 0.001
Stroke	206 (7.8%)	152 (6.1%)	54 (29%)	< 0.001
Liver condition	185 (7.3%)	170 (7.3%)	15 (6.8%)	0.837
Drugs				
Antihypertensive drugs	1679 (64%)	1523 (63%)	156 (76%)	0.003
IR indices				
TyG index	9.11 (8.70, 9.54)	9.09 (8.70, 9.53)	9.22 (8.66, 9.55)	0.448
TyG‐BMI index	294 (248, 345)	293 (248, 343)	302 (256, 366)	0.038
TG/HDL‐C ratio	2.80 (1.73, 4.28)	2.76 (1.72, 4.28)	3.15 (1.95, 5.04)	0.067
METS‐IR	51 (43, 60)	50 (42, 60)	54 (45, 64)	0.005

*Note:* Mean ± SD for continuous; *n* (%) for categorical. The percentages reported in this table are weighted statistics representing the proportions of the entire US population. Chi‐squared test with Rao & Scott's second‐order correction; Wilcoxon rank‐sum test for complex survey samples.

Abbreviations: BMI, body mass index; BUN, urea nitrogen; CHD, coronary heart disease; eGFR, estimated glomerular filtration rate; FPG, fasting glucose; HDL‐C, high‐density lipoproteincholesterol; LDL‐C, low‐density lipoprotein cholesterol; METS‐IR, metabolic score for insulin resistance; TC, total cholesterol; TG, total triglyceride; TG/HDL‐C, triglyceride to high‐density lipoprotein cholesterol ratio; TyG, triglyceride glucose; TyG‐BMI, triglyceride glucose with body mass index; UA, uric acid; UP, urine protein.

We assessed the potential bias caused by excluding patients with missing HF status by comparing the baseline characteristics, including the IR indices (TyG, TyG‐BMI, TG/HDL‐C, and METS‐IR), between patients with complete data and those excluded due to missing HF status. There were no statistically significant differences in the IR indices or other key variables between the two groups (*p* > 0.05 for all comparisons), suggesting that the missingness in HF status was likely random and unrelated to the key variables of interest. This indicates that excluding these patients did not introduce significant bias into the analysis.

### 3.2 Relationship Between IR Index and Occurrence of HF in Diabetic Patients

**FIGURE 2:**
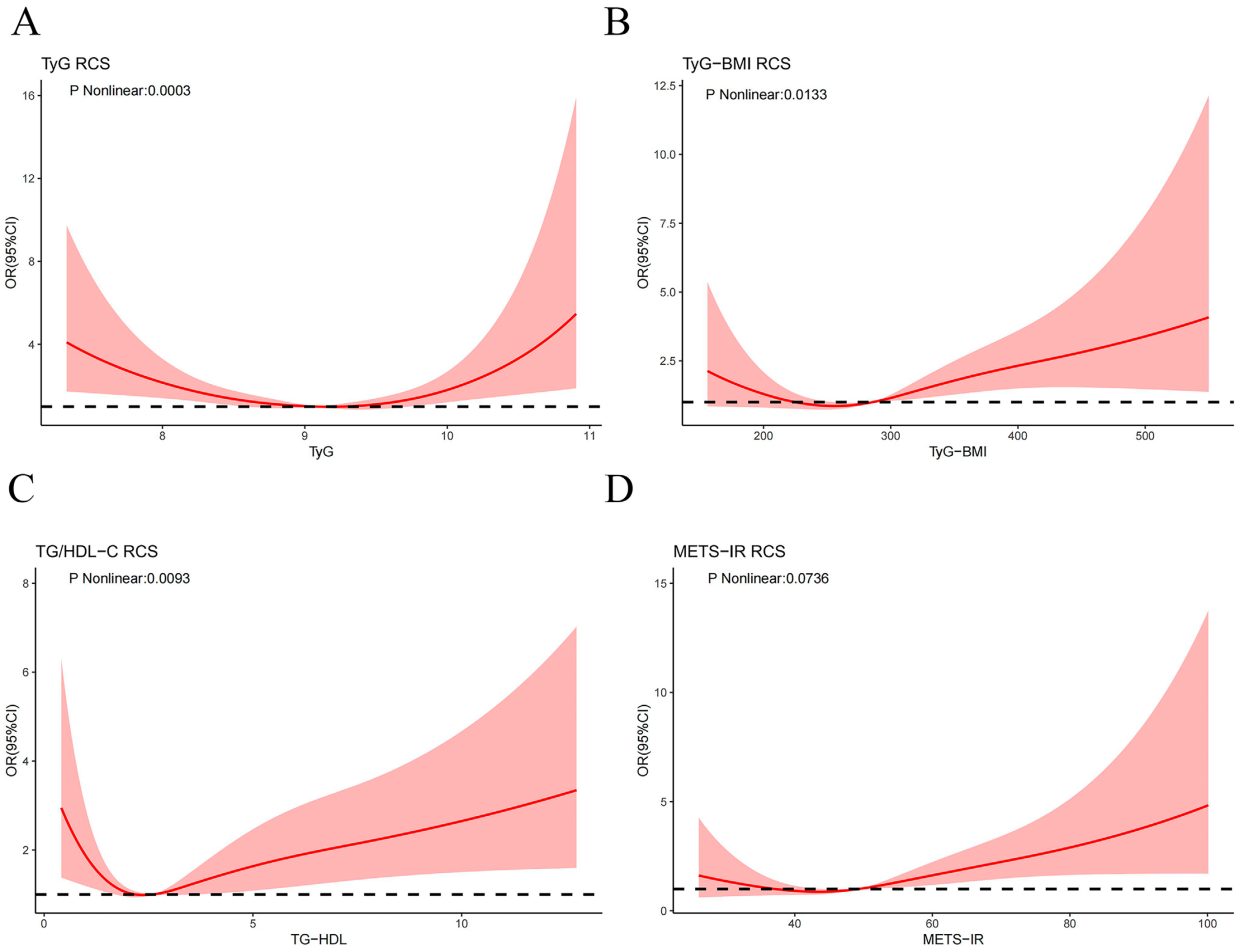
Restricted cubic spline curves for HF by four IR indices after covariate adjustment.

Table 2 delineates the complex relationship between the four IR indices and the incidence of HF in diabetic patients. In the unadjusted weighted one‐way logistic regression analysis (Model 1), TyG‐BMI (OR: 1.003, 95% CI: 1.000–1.005), TG/HDL‐C (OR: 1.090, 95% CI: 1.008–1.180), and METS‐IR (OR: 1.019, 95% CI: 1.006 ~ 1.032) demonstrated a significant association with the onset of HF in diabetic patients. In the fully adjusted Model 3, TyG‐BMI (OR: 1.005, 95% CI: 1.002–1.009), TG/HDL‐C (OR: 1.138, 95% CI: 1.024–1.265), and METS‐IR index (OR: 1.035, 95% CI: 1.015–1.057) emerged as significantly correlated factors with the development of HF in diabetes patients. As shown in Figure 2, there was a statistically significant u‐type dose–response correlation between TyG (nonlinear *p* < 0.001), TyG‐BMI (nonlinear *p* = 0.013), TG/HDL‐C (nonlinear *p* = 0.009), and the risk of HF in diabetic patients. Specifically, higher TyG (> 9.07), TyG‐BMI (> 225.22), and TG/HDL‐C (> 2.08) were associated with an increased risk of HF in diabetic patients. In contrast, among diabetic patients, the METS‐IR index was linearly correlated with the likelihood of HF, meaning that the risk of HF progressively increased with an increase in the METS‐IR index.

**TABLE 2:** The relationship between the four IR indices and the occurrence of HF in diabetic patients.

	Model 1	*p*	Model 2	*p*	Model 3	*p*
	OR (95% CI)		OR (95% CI)		OR (95% CI)	
TyG index	1.099 (0.805, 1.500)	0.600	1.339 (0.934, 1.920)	0.110	1.182 (0.791, 1.765)	0.400
TyG‐BMI index	1.003 (1.000, 1.005)	0.033	1.007 (1.004, 1.009)	< 0.001	1.005 (1.002, 1.009)	0.005
TG‐HDL‐C index	1.090 (1.008, 1.180)	0.031	1.136 (1.033, 1.249)	0.009	1.138 (1.024, 1.265)	0.017
METS‐IR	1.019 (1.006, 1.032)	0.005	1.041 (1.027, 1.055)	< 0.001	1.035 (1.015, 1.057)	0.001

*Note:* Model 1: unadjusted; Model 2: adjusted for age, gender, race, drink, and smoke; Model 3: Model 2 + further adjusted for physical activity (vigorous, moderate), dietary pattern (dietary protein intake, carbohydrate intake, total sugar intake, total fat intake, cholesterol intake), total cholesterol (TC), low‐density lipoprotein cholesterol(LDL‐C), urine protein (UP), uric acid (UA), urea nitrogen (BUN), estimated glomerular filtration rate (eGFR), the chronic disease (hypertension, stroke, CHD, liver condition), Antihyperglycemic drugs.

Abbreviations: CI, confidence interval; OR, odds ratio.

### 3.3 Predictive Performance of IR Indicators in the Risk Assessment of HF in Patients With Diabetes

**FIGURE 3:**
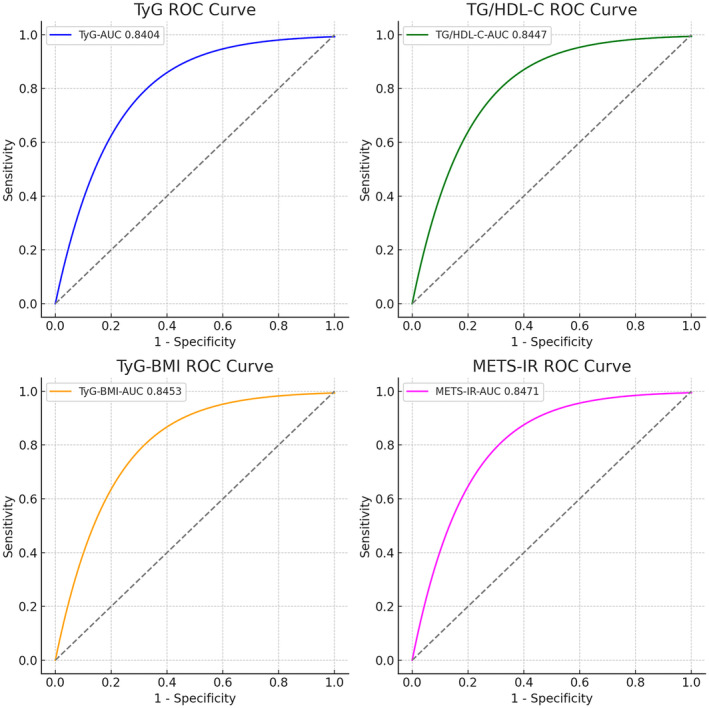
ROC for different IR surrogates to predict HF.TyG, triglyceride glucose; TyG‐BMI, triglyceride glucose with body mass index; TG/HDL‐C, triglyceride to high‐density lipoprotein cholesterol ratio; METS‐IR, metabolic score for IR.

ROC curves were meticulously constructed to assess the prognostic performance of the foundational model in patients with diabetes, adjusted for covariates such as gender, age, race, smoking history, alcohol consumption history, physical activity (vigorous, moderate), dietary pattern (dietary protein intake, carbohydrate intake, total sugar intake, total fat intake, cholesterol intake), total cholesterol (TC), low‐density lipoprotein cholesterol (LDL‐C), urine protein (UP), uric acid (UA), urea nitrogen (BUN), estimated glomerular filtration rate (eGFR), chronic disease (hypertension, stroke, CHD, liver condition), antihyperglycemic drugs. Additionally, four IR indices were incorporated into the prediction of HF risk (Figure 3). The findings (Table 3) revealed that the METS‐IR demonstrated the highest AUC of 0.847, followed by TyG‐BMI (AUC = 0.845), and TG/HDL‐C (AUC = 0.845). However, the DeLong test indicated that the enhancement of the predictive power of the baseline model by these four IR metrics was not significant. To assess the model's calibration, we performed the Hosmer‐Lemeshow goodness‐of‐fit test, which resulted in *p* > 0.05 for all models, indicating that the predicted probabilities aligned well with the observed outcomes (Table 4). This suggests that the models are not only good at discrimination but also exhibit satisfactory calibration.

**TABLE 3:** Main results of the ROC analysis.

	AUC	95% CI	*p*	Comparison *p*‐values
Base‐model	0.8405	0.8144–0.8665	< 0.001	Ref.
TyG index	08404	0.8143–0.8665	< 0.001	0.9049
TyG‐BMI index	0.8453	0.8197–0.8709	< 0.001	0.1068
TG‐HDL‐C index	0.8447	0.8193–0.8700	< 0.001	0.1675
METS‐IR	0.8471	0.8217–0.8725	< 0.001	0.0911

**TABLE 4:** Hosmer‐Lemeshow goodness‐of‐fit test.

Decile of risk	Observed HF events	Expected HF events	Observed non‐HF events	Expected non‐HF events	*χ* ^2^
1	2	2.4	98	97.6	0.05
2	4	3.9	96	96.1	0.07
3	5	5.2	95	94.8	0.10
4	7	6.8	93	93.2	0.05
5	8	8.1	92	91.9	0.12
6	9	9.4	91	90.9	0.08
7	10	10.3	90	89.7	0.05
8	11	11.2	89	88.8	0.06
9	12	12.5	88	87.5	0.05
10	14	13.9	86	86.1	0.09

### 3.4 Subgroup Analyses

**FIGURE 4:**
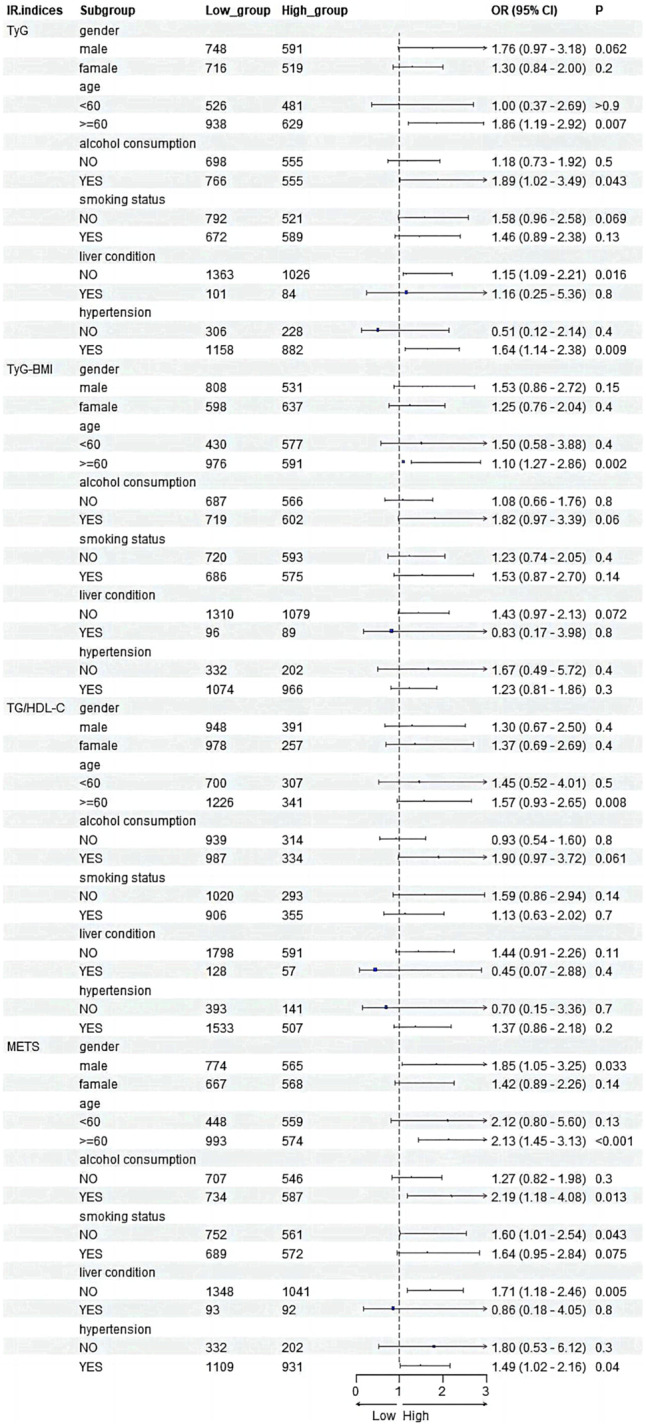
Association between IR indices and HF among diabetic patients in different subgroups. Each subgroup was adjusted for age, gender, drink, smoke, liver condition, and history of hypertension.

Subgroup analysis was conducted according to age, gender, alcohol consumption, smoking status, history of liver disease, and history of hypertension. As shown in Figure 4, the results showed that higher levels of all four IR indices were significantly associated with the risk of HF among diabetic patients aged 60 years or older. In addition, a higher TyG index was significantly associated with an increased risk of HF in subgroups such as alcohol drinkers (OR: 1.89, 95% CI: 1.02–3.49, *p* = 0.043), those without a history of liver disease (OR: 1.15, 95% CI: 1.09–2.21, *p* = 0.016) and those with a history of hypertension (OR: 1.64, 95% CI: 1.14–2.38, *p* = 0.009), compared to those with higher METS‐IR scores in men (OR: 1.85, 95% CI: 1.05–3.25, *p* = 0.033), alcohol drinkers (OR: 2.19, 95% CI: 1.18–4.08, *p* = 0.013), non‐smokers (OR: 1.60, 95% CI: 1.01–2.54, *p* = 0.043), those without a history of liver disease (OR: 1.71, 95% CI: 1.18–2.46, *p* = 0.005) and those without a history of hypertension (OR: 1.49, 95% CI: 1.02–2.46, *p* = 0.04). All four IR indices were significantly and positively associated with the risk of HF, especially in elderly patients with diabetes mellitus.

### 3.5 Sensitivity Analysis

In the conducted sensitivity analysis, the results demonstrated a general robustness (Table 5). We evaluated the influence of lipid‐lowering medications and the duration of diabetes on these outcomes. The adjusted findings revealed that the associations of TyG‐BMI (OR: 1.007, 95% CI: 1.001–1.012), TG/HDL‐C (OR: 1.307, 95% CI:1.166–1.464), and METS‐IR metrics (OR: 1.050, 95% CI: 1.020–1.081) remained significantly linked to the risk of developing HF in patients with diabetes.

**TABLE 5:** Key findings of the sensitivity analysis.

	Model 1	*p*	Model 2	*p*	Model 3	*p*
	OR (95% CI)		OR (95% CI)		OR (95% CI)	
TyG index	1.195 (0.803, 1.779)	0.400	1.554 (1.018, 2.372)	0.041	1.445 (0.895, 2.333)	0.130
TyG‐BMI index	1.004 (1.001, 1.007)	0.010	1.008 (1.005, 1.011)	< 0.001	1.007 (1.001, 1.012)	0.015
TG‐HDL‐C index	1.164 (1.067, 1.269)	< 0.001	1.232 (1.118, 1.359)	< 0.001	1.307 (1.166, 1.464)	< 0.001
METS‐IR	1.028 (1.013, 1.043)	< 0.001	1.054 (1.038, 1.071)	< 0.001	1.050 (1.020, 1.081)	0.001

*Note:* Model 1: unadjusted; Model 2: adjusted for age, gender, race, drink, and smoke; Model 3: Model 2 + further adjusted for physical activity (vigorous, moderate), dietary pattern (dietary protein intake, carbohydrate intake, total sugar intake, total fat intake, cholesterol intake), total cholesterol (TC), low‐density lipoprotein cholesterol (LDL‐C), urine protein (UP), uric acid (UA), urea nitrogen (BUN), estimated glomerular filtration rate (eGFR), the chronic disease (hypertension, stroke, CHD, liver condition), Antihyperglycemic drugs, lipid‐lowering drugs and diabetes duration.

## 4 Discussion

In this study, we investigated the association between several IR indices and HF occurrence in US adults with diabetes mellitus using the nationally representative NHANES dataset. TyG‐BMI, TG/HDL‐C, and METS‐IR indices were significantly associated with the risk of HF among patients with diabetes. Notably, the TyG, TyG‐BMI, TG/HDL‐C indices showed a significant U‐shaped dose–response relationship with the risk of HF among patients with diabetes. Additionally, all four IR indices were positively correlated with the risk of HF among older adults with diabetes. This study may be helpful for reducing the risk of HF in patients with diabetes.

### 4.1 Prior Evidence Elucidating the Relationship Between IR and HF

In the Cardiovascular Health Study, Banerjee et al. undertook a comprehensive 12‐year prospective investigation of 4425 participants, excluding those with myocardial infarction, and further narrowed their focus to 1216 individuals presenting with sudden heart failure. The study revealed a significant positive correlation between fasting insulin levels and the risk of developing HF (Banerjee et al. 2013). In a large prospective study of 56,149 participants, Zheng et al. reported that individuals with an elevated cumulative TyG index were at higher risk for HF during a median follow‐up of 10.04 years. The highest risk of developing HF was observed in the uppermost quartile group, and the multifactorial adjusted HR (95% CI) was 1.40 (1.15, 1.71) (Zheng et al. 2023). A Mendelian randomization study using a combined study population of 95,996 and 19,345 participants from Kailuan and Hong Kong, respectively, has found an independent association between an elevated TyG index and an increased risk of HF, and MR analysis suggests this association is causal (Li et al. 2022). Our findings are consistent with previous reports, and multivariate‐adjusted restricted cubic spline regression model revealed a nonlinear correlation between TyG index and incidence of HF in patients with diabetes mellitus (nonlinear *p* = 0.0001). In a retrospective cohort study conducted at a singular medical center, Zhang et al. found a significant association between METS‐IR and the incidence of MACEs in patients diagnosed with ICM in conjunction with T2DM. Notably, this correlation retained its significance even following meticulous adjustments for other pertinent confounding factors. The inclusion of METS‐IR in established risk prediction models had incremental accuracy in the prediction of MACEs (AUC = 0.637, 95% CI: 0.605–0.670, *p* < 0.001; NRI = 0.191, *p* < 0.001; IDI = 0.028, *p* < 0.001) 11. In an inquiry conducted by Wu et al., wherein three non‐insulin‐based IR indices were evaluated for their performance in forecasting the existence and extent of CAD, it was revealed that the TG/HDL‐C ratio, TyG index, and METS‐IR could be used as prognostic markers for both the occurrence and severity of CAD. Significantly, among these non‐insulin‐based IR indices, the METS‐IR index emerged as the most potent predictor, demonstrating the highest predictive capacity in this context (Wu et al. 2022). Similarly, in an investigation encompassing 546 patients diagnosed with HF combined with type 2 diabetes mellitus with a 12‐month follow‐up, Guo et al. revealed a positive correlation between the TyG index and the prognostic outcomes among HF patients accompanied by Type 2 DM. Notably, an escalated TyG index corresponded to an increased risk of cardiovascular mortality or rehospitalization attributable to HF in patients with chronic HF combined with DM (*p* < 0.05) (Guo et al. 2021). Miao et al. reported a prospective study involving 16,834 individuals, revealing that TyG and TyG‐related surrogate markers were significantly associated with hypertension and cardiovascular disease risk (Miao et al. 2024). Unlike the majority of previous studies, which primarily examined the correlation between individual IR index and the incidence of HF, this study offers a comprehensive evaluation. It specifically investigates the relationship between four distinct IR indices and the vulnerability to HF in diabetic patients. Furthermore, it conducts a comparative analysis of the prognostic value of these four IR indices for HF in diabetic patients.

### 4.2 Association Between Elevated IR Indices and HF Risk in the Old Diabetic Population

This study showed that all four IR indices were positively associated with the risk of HF in elderly patients with diabetes. This association may be due to the fact that aging is a well‐known risk factor for CHD, and the physiological characteristics of the elderly make them more susceptible to IR. IR reduces cellular insulin sensitivity and attenuates physiological insulin action, which leads to hyperglycemia. In addition, elderly patients with CHD are at higher risk of developing HF (Sciomer et al. 2020). Some studies have reported that HF accounts for a greater proportion of deaths in elderly T2DM patients (Huang et al. 2014; Clua‐Espuny et al. 2017). Since IR is considered an early stage of diabetes mellitus development, it should be noted that IR can be reversed by interventions. Our results suggest that IR indices may be used as potential predictors for clinical identification of elderly patients with diabetes at risk of heart failure, so that early intervention strategies can reduce the incidence of HF and improve prognosis.

Research indicates that individuals with type 1 diabetes mellitus (T1DM) face an increased risk of cardiovascular disease (CVD) and related complications (de Ferranti et al. 2014). IR can develop in T1DM patients due to intensive insulin therapy, weight gain, and metabolic disturbances, potentially exacerbating CVD risk (Polsky and Ellis 2015). T1DM patients with IR exhibit metabolic profiles similar to those with type 2 diabetes, suggesting shared mechanisms of cardiovascular dysfunction (Nadeau et al. 2010). Studies have shown that T1DM adolescents demonstrate reduced cardiopulmonary fitness, vascular reactivity, and diastolic function compared to non‐diabetic controls, with IR being a primary predictor of decreased peak oxygen consumption. The interplay between T1DM and IR is complex, involving factors such as autoimmune inflammation and long‐term insulin treatment effects. Management approaches for CVD risk reduction in T1DM have largely been extrapolated from T2DM experiences, despite important differences in disease duration and underlying pathophysiology (de Ferranti et al. 2014). Although our study did not specifically examine T1DM patients, the potential overlap between IR in T1DM and T2DM highlights the need for further research to clarify whether the relationships observed in T2DM populations are also applicable to T1DM.

### 4.3 IR's Mechanistic Impact on HF Development in Patients With Diabetes

The relationship between these IR parameters and HF in patients with diabetes can be partially explained through the following mechanistic pathways: (1) IR may elevate cardiac uptake and β‐oxidation of fatty acids beyond the capacity of mitochondrial oxidation. This results in mitochondrial dysfunction, leading to an increase in reactive oxygen species production. Consequently, elevated levels of reactive oxygen species reduce the oxidative capacity of fatty acids, further promoting mitochondrial dysfunction and apoptosis processes. This sequence ultimately leads to lipid accumulation, cardiac fibrosis, and the onset of HF (Jia, Whaley‐Connell, and Sowers 2018; Lee 2022). (2) IR significantly contributes to the pathogenesis of HF by augmenting the accumulation of unfolded proteins, which triggers endoplasmic reticulum stress. Concurrently, IR suppresses Ca^2+^ ATPase activity by reducing glucose uptake in cardiac tissues, leading to increased intracellular Ca^2+^ concentrations. This surge in intracellular Ca^2+^ content prompts the opening of the mitochondrial membrane permeability transition pore, ultimately causing energy deficiency and promoting oxidative stress in HF. Conversely, endoplasmic reticulum stress impedes insulin signaling pathways by triggering pro‐inflammatory kinases such as c‐Jun amino‐terminal kinase and protein kinase C. The interplay between endoplasmic reticulum stress and abnormal calcium handling enhances apoptosis, necrosis, and autophagy processes in cardiomyocytes, ultimately leading to HF (Henstridge, Whitham, and Febbraio 2014; Rupee et al. 2023). (3) IR augments the activation of the renin‐angiotensin‐aldosterone system, achieved through the upregulation of angiotensinogen, angiotensin II, and its receptor. Concurrently, angiotensin II suppresses the phosphatidylinositol 3‐kinase pathway while simultaneously stimulating the mTOR/S6K1 signaling pathway. This results in systemic and cardiac IR. The interplay between these processes contributes to myocardial fibrosis, cardiac diastolic dysfunction, and the onset of HF (Adams Jr. 2004; Verbrugge, Tang, and Mullens 2015; Jia et al. 2015). (4) IR can disrupt glycolipid metabolism, a process that can be triggered by non‐enzymatic glycosylation to produce AGEs. These AGEs directly stimulate the cross‐linking of connective tissues, leading to the development of vascular sclerosis. Additionally, AGEs can enhance the deposition of extracellular matrix proteins through the activation of the nuclear factor‐κB signaling pathway. Simultaneously, they amplify inflammatory responses by increasing the production of ROS, ultimately resulting in myocardial fibrosis (Zhao, Randive, and Stewart 2014; Jia, DeMarco, and Sowers 2016). (5) IR is observed to have a simultaneous association with metabolic disorders and subclinical, low‐grade inflammation. The disruptions in metabolic processes trigger subcellular, low‐grade inflammation within the myocardium, leading to cardiomyocyte damage, apoptotic events, and the progression of cardiac fibrosis. Consequently, this results in impaired diastolic and systolic functions of the heart (Jia et al. 2015).

The nonlinear, U‐shaped relationship between certain IR indices and the risk of HF observed in our study, suggests a complex interplay between metabolic factors and cardiovascular health. This pattern could reflect the dual nature of IR and its varying effects on different biological processes, depending on its severity. Several potential mechanisms might explain this U‐shaped association. At low levels of IR, compensatory mechanisms may provide protection against metabolic dysregulation. For instance, the body may enhance insulin sensitivity in peripheral tissues to maintain glucose homeostasis. However, at very low levels of IR, this compensatory action might overstimulate certain pathways, leading to metabolic imbalances such as impaired fatty acid oxidation, abnormal energy metabolism, or oxidative stress, all of which could increase cardiovascular risk (Kolb et al. 2020). Conversely, at high levels of IR, the harmful effects of IR become more pronounced, contributing to the pathogenesis of HF. Elevated IR can promote inflammation in cardiomyocytes, mitochondrial dysfunction (Perticone et al. 2019; Tajes et al. 2021), and increased oxidative stress (Cadenas 2018). These mechanisms can induce cardiac fibrosis, inflammation, and cellular apoptosis, ultimately leading to structural and functional damage in the heart. Increased mitochondrial dysfunction and endoplasmic reticulum stress associated with high IR levels may further impair the heart's capacity to manage metabolic stress, thus elevating HF risk (Kriebel, Vautrin, and Holsapple 1990). The identification of a U‐shaped relationship between IR indices and HF risk may have significant clinical implications. It raises the possibility of defining a “safe” or “optimal” range for IR indices, within which HF risk is minimized. Understanding this relationship could guide the early detection of patients at risk for HF and allow for timely interventions aimed at maintaining IR within this optimal range. Interventions could include lifestyle modifications, pharmacological strategies, or both, to prevent patients from crossing into higher‐risk IR levels. Future studies should aim to establish clear thresholds for these indices to inform clinical decision‐making.

### 4.4 Strengths and Limitations

The data used in this study were collected from a nationally representative sample of US adults. This increases the statistical power and strengthens the validity of the results. We investigated the relationship between four IR metrics and HF incidence in patients with diabetes. Furthermore, we evaluated the prognostic value of each of the four IR indices for predicting the risk of HF in patients with diabetes. However, several limitations should be noted. Firstly, while this study has identified a correlation between IR indices and HF status in diabetic patients via cross‐sectional analysis, it is crucial to note that the findings do not endorse the utilization of these indices for prospective identification of HF risk in pre‐diabetic patients. Future research should focus on devising effective methods to predict HF risk during the pre‐diabetic phase or even prior to diabetes onset. In clinical practice, identifying predictive tools for HF risk before diabetes onset and potentially even before the onset of HF is paramount. This will be further investigated in future studies with the aim of intervening as early as possible. Secondly, the potential presence of recall bias in the retrospective data used in our study must be acknowledged. Thirdly, due to missing data on TyG and TyG‐related indicators, diabetes mellitus, and HF, some patients were excluded from our study, significantly reducing the original cohort size. Thus, both of these factors may have contributed to selection bias. Fourth, HF‐related data were self‐reported and patients with undiagnosed HF were excluded, which could have resulted in an underestimation of the true prevalence of heart failure. In addition, we included as many clinically relevant variables as possible to minimize confounding in multivariate analyses; however, confounding remains a possibility. Finally, our study was conducted in the United States; further research is needed to confirm these findings in other geographic regions.

## 5 Conclusion

This study posits a potential correlation between indicators of IR and the incidence of diabetes mellitus‐related HF among US adults. This association may serve as a significant predictor for the risk of HF in patients with diabetes mellitus. However, its applicability to prospectively identify the risk of HF in non‐HF patients with diabetes mellitus warrants further exploration.

## Author Contributions

L.C. and L.Q. conceived of the study, and Y.L. participated in its design and coordination, and L.C. draft the manuscript. All authors read and approved the final manuscript.

## Ethics Statement

The authors have nothing to report.

## Consent

The authors have nothing to report.

## Conflicts of Interest

The authors declare no conflicts of interest.

## Data Availability

The original contributions presented in the study are included in the article, further inquiries can be directed to the corresponding author.
